# Longitudinal investigation of natural killer cells and cytokines in chronic fatigue syndrome/myalgic encephalomyelitis

**DOI:** 10.1186/1479-5876-10-88

**Published:** 2012-05-09

**Authors:** Ekua W Brenu, Mieke L van Driel, Donald R Staines, Kevin J Ashton, Sharni L Hardcastle, James Keane, Lotti Tajouri, Daniel Peterson, Sandra B Ramos, Sonya M Marshall-Gradisnik

**Affiliations:** 1 Faculty of Health Science and Medicine, Population Health and Neuroimmunology Unit, Bond University, Robina, QLD, Australia; 2 Faculty of Health Science and Medicine, Bond University, Robina, QLD 4229, Australia; 3 School of Medical Science, Griffith Health Institute, Griffith University, Gold Coast Campus, Gold Coast, QLD, Australia; 4 Discipline of General Practice, School of Medicine, The University of Queensland, Brisbane, Australia; 5 Queensland Health, Gold Coast Public Health Unit, Robina, Gold Coast, QLD, Australia; 6 Sierra Internal Medicine, Incline Village, Nevada, USA

**Keywords:** Chronic fatigue syndrome, Cytokines, Cytotoxic activity

## Abstract

**Background:**

Chronic Fatigue Syndrome/Myalgic Encephalomyelitis (CFS/ME) is an etiologically unexplained disorder characterised by irregularities in various aspects of the immunological function. Presently, it is unknown whether these immunological changes remain consistent over time. This study investigates Natural Killer (NK) cell cytotoxic activity, NK cell subsets (CD56^bright^CD16^-^ and CD56^dim^CD16^+^) and cytokines, over the course of a12 month period in patients with CFS/ME.

**Methods:**

The participants in the study comprised 65 (47.2 ± 11.5 years) CFS/ME participants and 21 (45.2 ±9.3 years) non-fatigued controls. Flow cytometry protocols were used to assess NK subsets and NK cytotoxic activity at various time points that included baseline (T1), 6 (T2) and 12 months (T3). Cytokine secretions were measured following mitogenic stimulation of peripheral blood mononuclear cells.

**Results:**

NK cytotoxic activity was significantly decreased in the CFS/ME patients at T1, T2 and T3 compared to the non-fatigued group. Additionally, in comparison to the non-fatigued controls, the CFS/ME group had significantly lower numbers of CD56^bright^CD16^-^ NK cells at both T1 and T2. Interestingly, following mitogenic stimulation, cytokine secretion revealed significant increases in IL-10, IFN-γ and TNF-α at T1 in the CFS/ME group. A significant decrease was observed at T2 in the CFS/ME group for IL-10 and IL-17A while at T3, IL-2 was increased in the CFS/ME group in comparison to the non-fatigued controls. Overall cytotoxic activity was significantly decreased at T3 compared to T1 and T2. CD56^bright^CD16^-^ NK cells were much lower at T2 compared to T1 and T3. IL-10 and IL-17A secretion was elevated at T2 in comparison to T1 and T3.

**Conclusion:**

These results confirm decreases in immune function in CFS/ME patients, suggesting an increased susceptibility to viral and other infections. Furthermore, NK cytotoxic activity may be a suitable biomarker for diagnosing CFS/ME as it was consistently decreased during the course of the 12 months study.

## Introduction

Immune responses to infection and inflammation are important aspects of physiological homeostasis. This involves constant co-ordinated responses from and between the innate and adaptive immune systems [1,2]. Cells of the innate immune system in particular Natural Killer (NK) cells are important mediators of targeted cell killing of tumor, transformed and virus infected cells [3]. NK cells are recruited by interferons and chemoattractive chemokines including CCL22, CX3CL1 and CXCL8 [4-7]. At sites of infection, stochastic expression of NK receptors with the release of granzymes and perforin via mitogenic pathways ensures efficient elimination of unwanted cells [8-11].

As a disease with unknown pathomechanism and lacking specific diagnostic markers, Chronic Fatigue Syndrome/Myalgic Encephalomyelitis (CFS/ME) has been associated with diminished immune function [12]. CFS/ME is characterised by severe fatigue with apparent flu-like symptoms that either fluctuate or deteriorate and persist for many months to years [13,14]. To date cytokines, lymphocyte subsets and cytotoxic activity, have been examined in patients with CFS/ME using serum, plasma or blood samples. The findings from these investigations demonstrate equivocal quantities in lymphocyte numbers and cytokines but interestingly consistent decrease in NK cytotoxic activity [15-20].

A decrease in NK cytotoxic activity is thus a recurring finding in CFS/ME research [16,17,20-24] and remains a hallmark of the disease. Additionally, equivocal changes in NK cell subsets, CD56^dim^CD16^+^ and CD56^bright^CD16^-^ NK cells have been shown to occur [20,25]. Abnormalities in cellular levels of either CD56^dim^CD16^+^ NK or CD56^bright^CD16^-^ NK cells can affect cytokine production and subsequent clearance of pathogens. Despite these striking alterations there are no follow up studies reporting on the profiling of both NK cells subsets and activity during an extended course of CFS/ME. Perturbations in cytokine production favouring either an anti-inflammatory or pro-inflammatory cytokine profile may be prevalent in some CFS/ME patients [26-28]. CFS/ME is a chronic disease with a relatively long duration, persisting for more than 6 months, however, it is not reported yet whether alterations in cytokines occur and persist over time in adults with CFS/ME.

At this stage the diagnosis of CFS/ME is based on self-reported clinical symptoms. The difficulty in establishing a stringent medical diagnosis for CFS/ME may be related to the lack of data monitoring the stability of immune markers during the course of the disease. Almost all studies to date investigating immune function in CFS/ME including cell activity, lymphocyte subsets and cytokines in CFS/ME adults were only restricted to single time point examination, providing insufficient information on the changes in these makers as the disease progresses. Additionally, there are many factors that can affect the differences in data reported. As CFS/ME is heterogeneous and multi-factorial, CFS/ME patients may experience periods of high, medium or low severity in symptoms which may be related to the levels of cytokines and immune function.

Therefore, the purpose of this study is to examine the validity and stability of immune parameters previously known to be compromised in CFS/ME and to determine most importantly whether these observations are consistent during the course of the disease. Assessment and evaluation of these markers potentially enables both the determination and knowledge of their stability over the course of the disease. This is the first longitudinal study assessing NK cytotoxic activity, NK subsets and CD4^+^T cell cytokine distribution over a period of 12 months in adults suffering from CFS/ME.

## Methods

### Recruitment

Participants for this study were comprised of CFS/ME patients and non-fatigued controls recruited from an existing cohort in Queensland and New South Wales, Australia [25]. Prior to inclusion all participants completed a consent form and a questionnaire. The description of these participants is provided in Table 1. The CFS/ME group met the 1994 CDC criteria for CFS/ME and the control group consisted of non-fatigued volunteers. The non-fatigued controls were recruited from similar locations as the CFS/ME cohort. In both groups, individuals with known autoimmune disorders, psychosis, epilepsy, diabetes and cardiac related disorders prior to the onset of CFS/ME like symptoms were excluded from the study. These exclusions were also applied to the control group.

**Table 1:** Baseline clinical characteristics of chronic fatigue syndrome patients (cases) and non-fatigued controls

	**Cases (n=65)**	**Controls (n=21)**
Age, mean in years (SD)	47.2 (11.5)	45.2 (9.3)
Female (%)	75.4	66.7
BMI kg/m^2^ (SD)	24.4 (4.9)	25.3 (5.5)
Smoked in the past 2 years (%)	4.7	4.8
**Symptom (%)**		
Weakness >24 hours after exercise	93.8	9.5
Unrefreshing sleep	93.8	19.0
Impaired concentration	90.8	4.8
Muscle pain	81.5	14.3
Joint pain	70.8	9.5
Headaches	67.7	9.5
Lymph glands	43.1	0
Sore throat	46.2	8.0
Diagnosed with depression or anxiety	64.6	19.0

### Data collection

Samples were collected at baseline (T1), after 6 months (T2) and after 12 months (T3). Blood collections were performed at three testing sites as it was not always feasible for participants to travel to the main testing site. Participants were recruited in 2009 and enlisted for the study in December 2009. The first collection was performed in December 2009. The second sampling point took place in June 2010 and the third in December 2010. Clinical data were collected through self-administered questionnaires at (T1) and T3.

### Sample preparation and routine measurements

Non-fasting morning blood samples were collected from the antecubital vein of all participants into lithium heparin (12 mL) and EDTA (25 mL) tubes. Regular full blood count (Coulter Counter, Beckman Coulter) assessments were performed ahead of immunological assessments. All analyses and experiments were performed immediately following blood collection. On a given day blood samples were collected from a maximum of 10 participants. These participants comprised of a mixture of CFS/ME patients and non-fatigued controls, however, as there were more CFS/ME patients compared to non-fatigued controls, in some cases the samples comprised only CFS/ME patients.

### NK cytotoxic activity

NK cytotoxic activity was examined as previously described [20,25,29]. In brief, NK lymphocytes were isolated from blood samples using density gradient centrifugation and labelled with 0.4% PKH-26 (Sigma, St Louis, MO). Following which NK cells were incubated with K562 cells, for 4 hours at 37°C in 95% air, 5% CO_2_ at an effector to target ratio of 25 (NK cells):1 (K562). An E:T ratio of 25:1 was chosen as we have previously found this ratio to be the most optimal condition for assessing cytotoxic activity. In previous studies this ratio has been used [20,25]. After four hours of incubation NK lysis of K562 cells was calculated as previously described [29] to determine the ability of NK cells to induce tumor cell death or apoptosis via FACS-Calibur flow cytometry (BD Bioscience, San Jose, CA), using Annexin V-FITC and 7-AAD reagents (BD Pharmingen, San Diego, CA). The NK assay was performed within 2–4 hours upon receipt of all blood samples for that particular day hence each sample was treated the same. Each sample was performed in duplicates and a control sample was included in each run.

### NK subsets

The frequency of NK cell subsets was evaluated as previously described [20,25]. Briefly, a negative selection system using RosetteSep Human Natural Killer Cell Enrichment Cocktail (StemCell Technologies, Vancouver, BC) was used to segregate NK lymphocytes from whole blood. Preferentially isolated NK cells were then labelled with CD56-FITC and CD16-PE monoclonal antibodies (BD Pharmingen, San Jose, CA). In a forward scatter and side scatter plot created using the FACS-Calibur flow cytometry, a lymphocyte gate was set on NK cells. Subset profile was measured in a PE versus FITC plot.

### T cell specific cytokine distribution

Isolated PBMCs were cultured at 1×10^6^ cells/mL with or without 1 μg of phytohemagluttinin for 72 hours. Cellular supernatants were collected following incubation and stored at −80°C for later assessment. Th1, Th2 and Th17 cytokine concentrations were determined using the cytometric bead array (CBA) kit (BD Pharmingen, San Jose, CA) [25,30]. The cytokines that were measured include IL-2, IL-4, IL-6, IL-10, tumour necrosis factor (TNF)-α, interferon (INF)-γ and IL-17A. The CBA kit includes a standard that is diluted at different concentrations as per manufacturer’s instructions to produce a standard curve for each cytokine to be measured. This was included in the cytokine assessment. During each run of the CBA analysis control samples were included and each set of samples analysed on a given day consisted of both CFS/ME patients and non-fatigued control cell supernatants.

### Statistical analysis

Statistical analysis was performed using SPSS software version 18.0. The experimental data represented in this study are reported as means plus/minus standard error of the mean (±SEM) while all the clinical date are reported as means plus/minus standard deviation (±SD). Comparative assessments among participants (CFS/ME and non-fatigued controls) were performed with the analysis of variance test (ANOVA) and repeated measures. For the repeated measures assessment time was the within-subjects factor and group was the between subject factor. Instances where Mauchly’s test indicated that the sphericity assumption was breached, the Huynh-Feldt correction was applied. The Bonferroni method was used as post-hoc analysis to assess changes in the data. Time effects and group effects on the variables measured were assessed using eta squared (η^2^), where η^2^ is the ratio of the sum of squares of the variance to the sum of squares of the total variance (η^2^ = SS_variance effect_/SS_total_). To determine within subject stability Pearson and Spearman’s rank correlations were determined between the three time points. P-values less than or equal to 0.05 were considered statistically significant.

### Ethical clearance and participant selection

Approval for this study was granted after review by the Bond University Human Research Ethics Committee (R0852A).

## Results

### Participants

Data for baseline, 6 months and 12 months were available for 86 participants (65 CFS/ME patients and 21 non-fatigued controls). The mean age for the CFS/ME patients was 47.2 ± 11.5 years and 45.2 ± 9.3 years for the non-fatigued controls. 75.4% of CFS/ME patients and 66.7% of the non-fatigued controls were female and the mean BMI in both groups was 24.5 ± 5.0 for the CFS/ME patients and 24.5 ± 4.3 for the non-fatigued controls. The CFS/ME patients had been suffering from fatigue for a mean of 16.4 ± 12.5 years and their mean score on the Fatigue Severity Scale (FSS) was 55.5 ± 9.1 (the lowest possible score of the FSS was 7 and highest is 63) [31]. Only 19.4% of patients indicated that they were still able to carry out normal activities. Clinical baseline characteristics are reported in Table 1.

### Longitudinal assessment of NK cytotoxic activity

**Figure 1:**
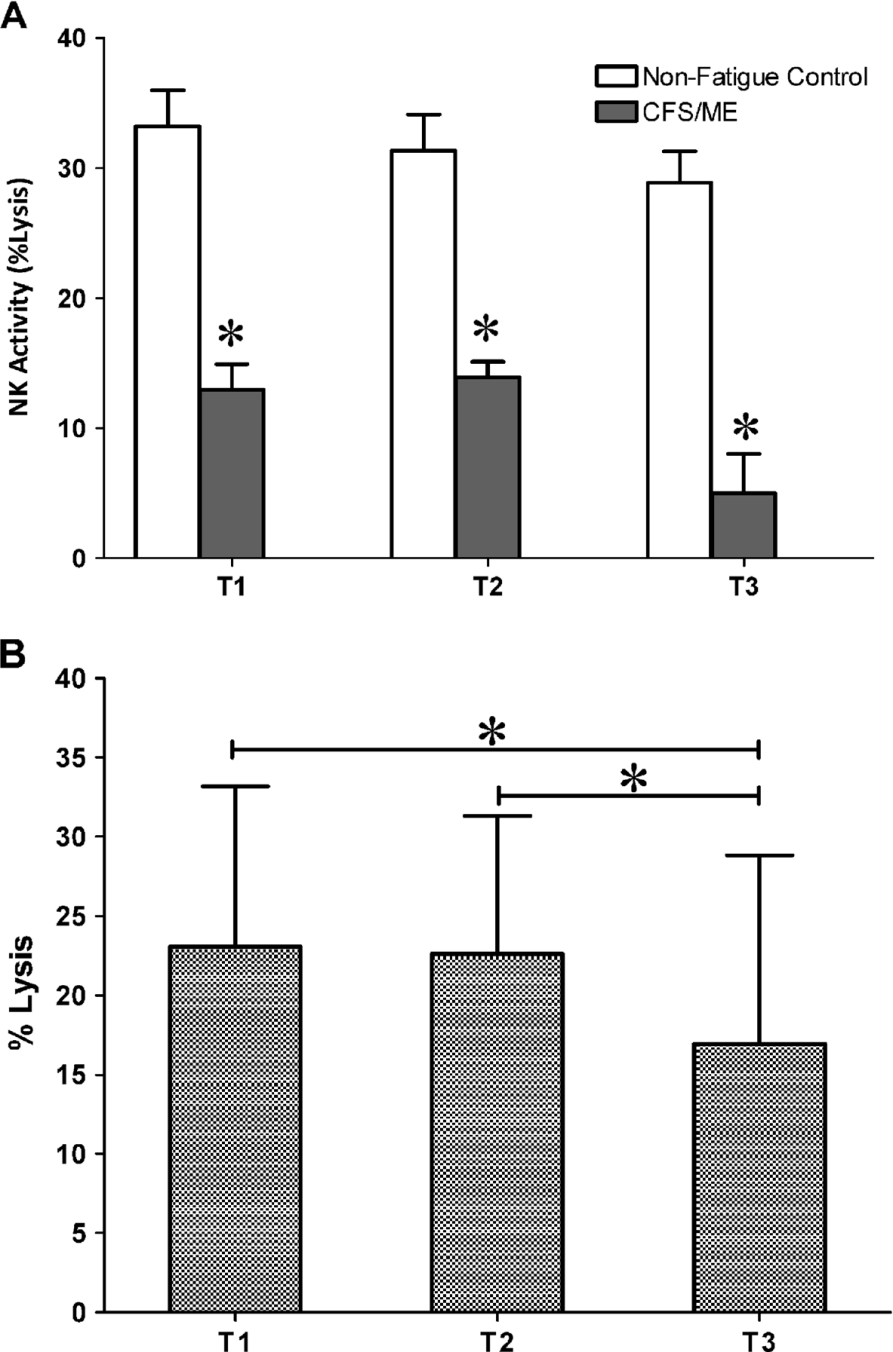
**NK Cytotoxic activity was decreased at all time points in the CFS/ME patients.(A)** Cytotoxic activity presented as % lysis of K562 cells by NK cells assessed overtime at T1, T2 and T3 in the CFS/ME patients (black bars) and control (white bars) participants. **(B)** Cluster analysis showing the overall cytotoxic activity in the whole participant group. *Indicates statistical significant results relative to controls. Statistics are presented as mean ± SEM.

NK cytotoxic activity, that is, the ability of the NK cells to effectively cause apoptosis of K562 cells was significantly reduced (*p <* 0.05) in CFS/ME patients compared to the control group. Significant changes between the groups were noticed at the different time points, i.e. at T1 (*p* < 0.001), T2 (*p* < 0.001) and T3 (*p* < 0.001) (Figure 1A). Regardless of the time point cytotoxic activity remained significantly decreased (*p* < 0.001). Time effects had no interaction with the group (*p* = 0.495). There were significant time effects cytotoxic activity from T1 to T3 (*p* = 0.023) and from T2 to T3 (*p* = 0.016) (Figure 1B).

### Differential distribution of NK cells between groups

**Figure 2:**
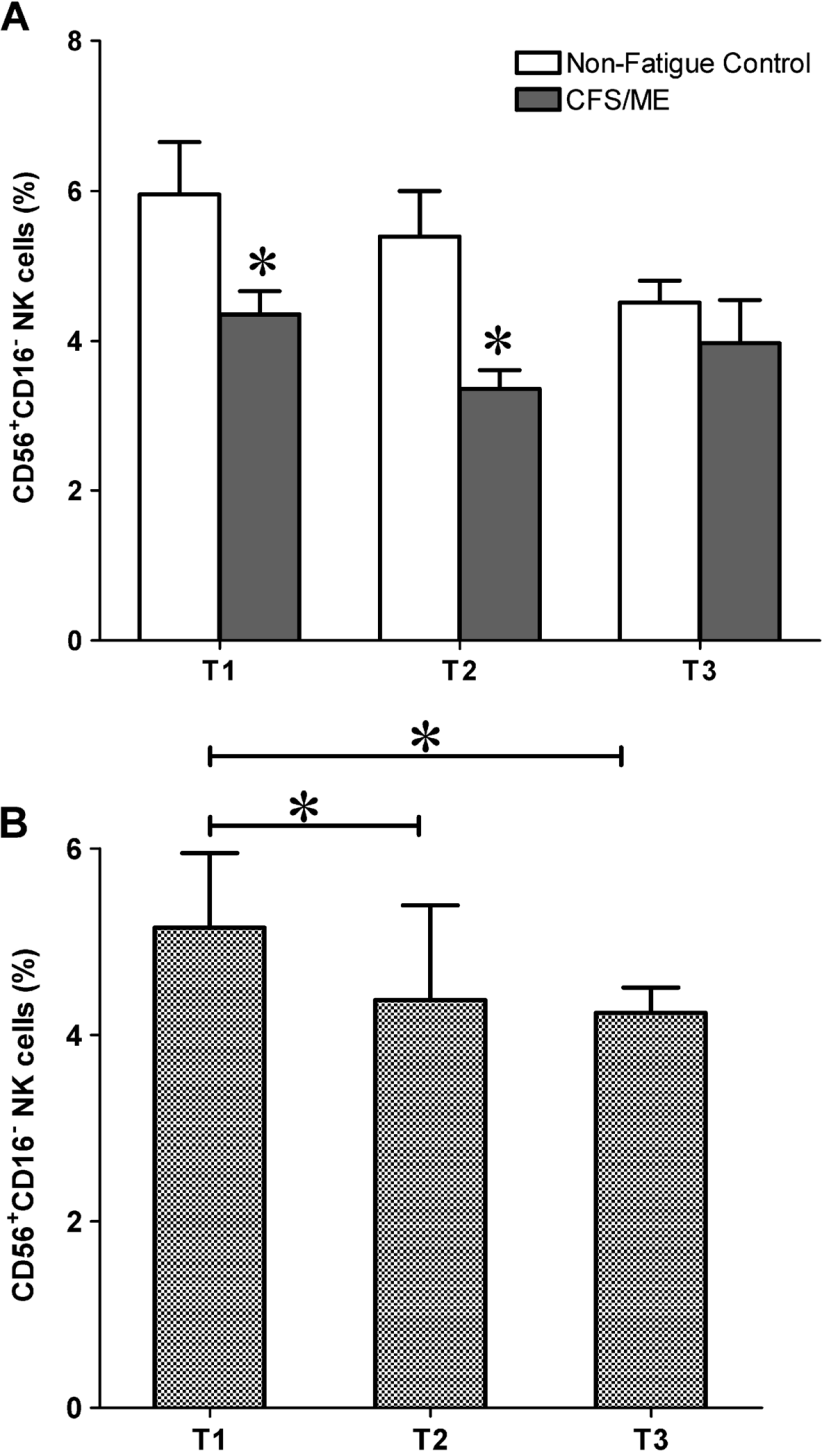
**CD56**^**bright**^**CD16**^**-**^**NK Subset was decreased in the CFS/ME group.** Percentage of NK cells stained positive for CD56^bright^CD16^-^ was significantly low in the CFS/ME group at the T1 and at T2. The white bars represent control data while the black bars represent CFS/ME data. **(B)** Cluster analysis showing the overall cytotoxic activity in the whole participant group. Data are presented in the form of log of the total events collected via flow cytometery ± SEM. *Signifies statistical significant results relative to controls.

NK subsets were classified as CD56^dim^CD16^+^ and CD56^bright^CD16^-^ NK cells. CD56^bright^CD16^-^ NK cells were significantly lower at T1 (*p* = 0.020) and T2 (*p* < 0.001) in CFS/ME patients compared to non-fatigued controls (Figure 2A). Significant time effects were observed for CD56^bright^CD16^-^NK cell (*p* = 0.003) in the overall group. Additionally, there was a significant interaction between time and group (*p* = 0.015). Pairwise comparison revealed significant changes in the overall group from the T1 to T2 (*p* = 0.037) and from T1 to T3 (*p* = 0.014) (Figure 2B). CD56^dim^CD16^+^NK cells remained unchanged throughout the study for both groups.

### T cell related cytokine distribution

**Figure 3:**
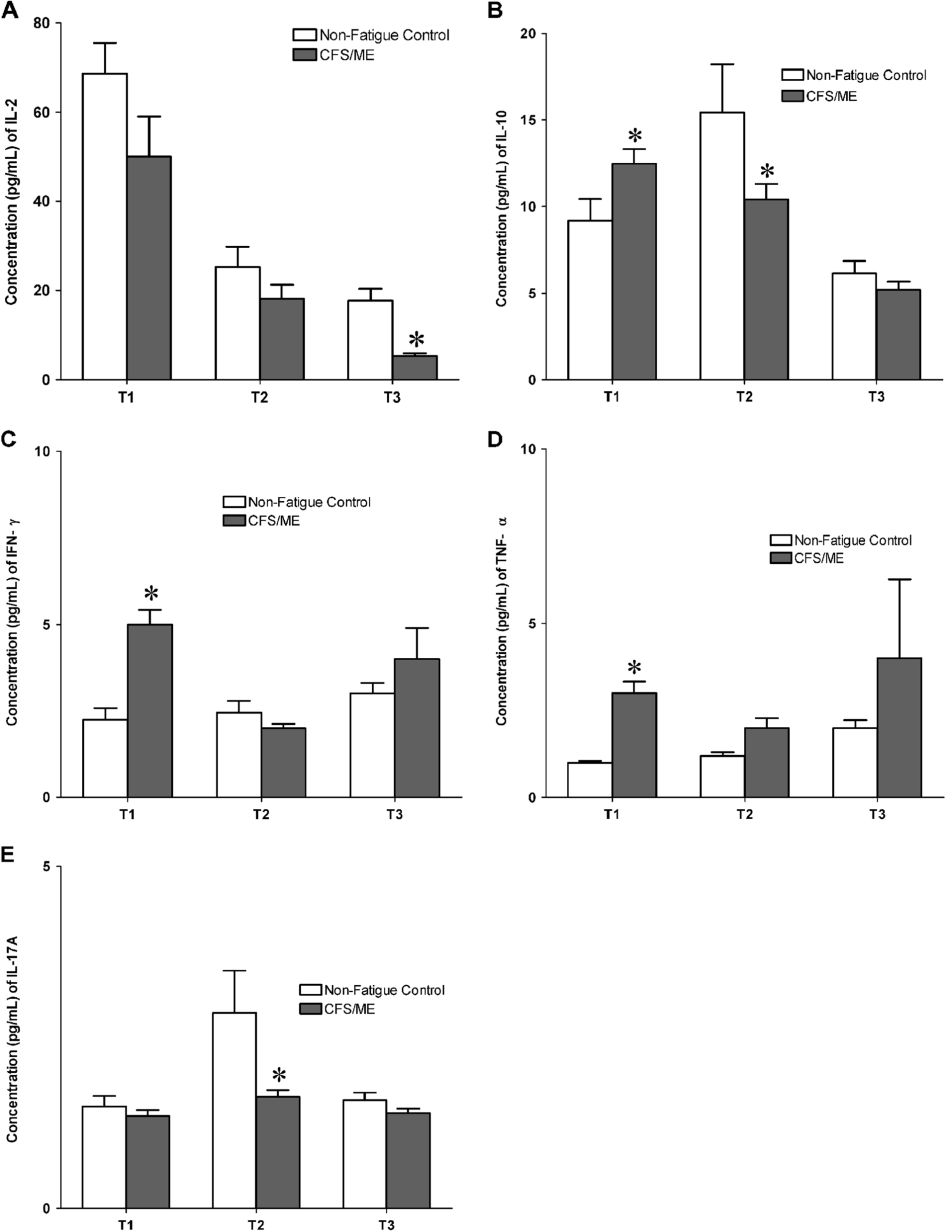
**Between group differences in cytokine production overtime.** The secretion of IL-2 **(A)** was significantly increased in the CFS/ME patients at the T3, **(B)** IL-10 increased at the T1 and dropped significantly at the T2 in the CFS/ME group. **(C)** IL-17A was reduced at the T1 while **(D)** IFN-γ and **(E)** TNF-α were increased significantly only at the T1. The CFS/ME data are signified by the black bars and non-fatigued controls the white bars. *Symbolizes statistically significant results were considered where p ≤ 0.05. The results are expressed as the mean concentration at each time point ± SEM.

Mitogenic stimulation produced significant differences in cytokine distribution observed at T1, T2 and T3. At T1, IL-10 (*p* = 0.051), IFN-γ (*p* = 0.003) and TNF-α (*p* = 0.002) were significantly increased in the CFS/ME patients compared to the non-fatigued controls (Figure 3B, D, E). IL-10 (*p* = 0.026) and IL-17A (*p* = 0.002) were significantly decreased at T2 (Figure 3B, C) and only IL-2 (*p* = 0.005) was significantly increased at T3 (Figure 3A). There was no significant difference in the remaining cytokines.

**Figure 4:**
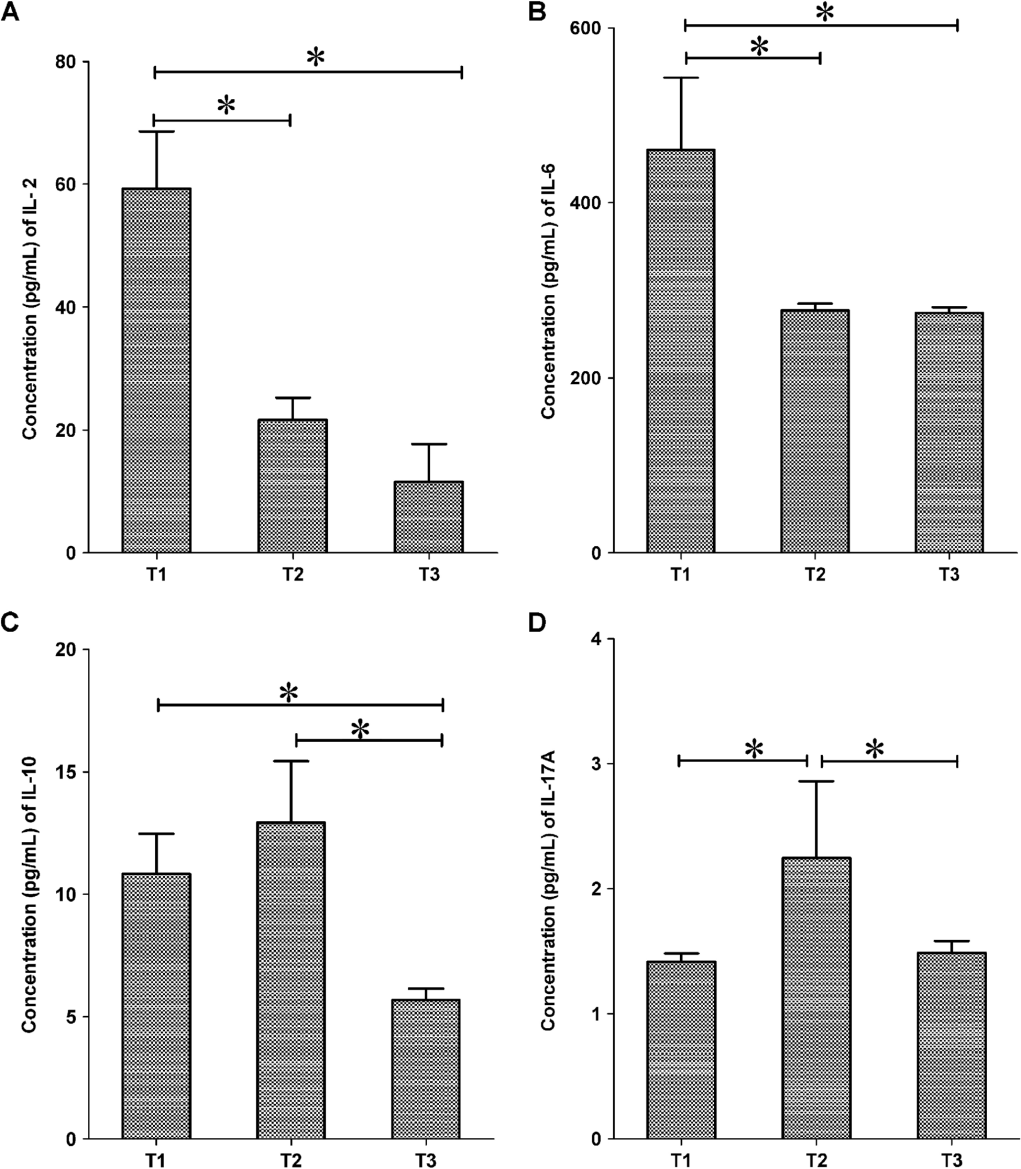
**Overall Cytokine Secretion with respect to time.(A)** Cytokine production within group was significantly different in IL-2 with high levels noticed at T1 compared to the T2 and T3 months. **(B)** A similar pattern was noticed in IL-2. **(C)** IL-10 was however significantly decreased at T3 compared to the T1 or at T2. **(D)** IL-17A tended to be higher at T2 within group. Data are presented in the form of log of the total events collected via flow cytometery ± SEM. *Designates statistical significant results relative to controls.

The production of some cytokines was significantly different from one time point to another. IL-2 was increased from T1 to T2 (*p* < 0.001) and from T1 to T3 (*p* < 0.001) (Figure 4A). IL-17A was increased from T1 to T2 (*p* = 0.009) but declined from T2 to T3 (p = 0.022) (Figure 4D). Secretion of IL-6 was decreased from T1 to T2 (*p* = 0.001) and T1 to T3 (*p* = 0.001) (Figure 4B). IL-10 progressively declined from T1 to T3 (*p* < 0.001) and T2 to T3 (*p* <0.001) (Figure 4C).

### Parameter stability

In this study the stability of the immune parameters over time was assessed using Pearson and Spearman’s correlation analysis, where significance was set at *P* less than or equal to 0.05. The results were highly significant for NK activity data (T1-T2; *p* = 0.003, T1-T3; *p* = 0.032). Although, CD56^bright^CD16^-^NK cells were not significantly decreased at T3 the correlation between time points was significant (T1-T2; *p* < 0.0001, T1-T3; *p* < 0.0001, T2-T3; *p* < 0.0001). Of note, cytokines were not correlated at any time point. However decreases in IFN-γ, TNF-α and IL-10 were correlated at T1.

## Discussion

Our investigation is the first study to demonstrate that NK cytotoxic activity remains consistently decreased in CFS/ME patients during the course of the disease. However, other immune parameters, especially cytokine secretions fluctuate at different time points and therefore demonstrate inconsistencies in their distribution pattern during the course of the disease. The purpose of this investigation was to identify immune markers that can be possibly used as biomarkers for CFS/ME in a longitudinal manner.

Primarily, this longitudinal study has illustrated that NK cytotoxic activity is potentially useful as a biomarker for CFS/ME, since it was the most stable in the CFS/ME patients over the 12 months period of the study. Decreases in cytotoxic activity occur in CFS/ME and in some cases this is associated with differential expression in the levels of cytotoxic molecules [20,21,32,33]. These cytotoxic proteins and their genes including perforin (*PRF1*), granzyme A (*GZMA*), granzyme B (*GZMB*) and granzyme K (*GZMK*) have been shown to be either increased or decreased in CFS/ME [24,25,34]. For example, the perforin gene (*PRF1*) may increase in some cases of CFS/ME while intracellular perforin protein may be decreased in other CFS/ME patients [25]. Such profound differences in protein and mRNA can reduce the effectiveness of NK cells to induce lysis/cytotoxic activity of viral infected cells. Hence, defective cytotoxic activity in CFS/ME patients may be due to perturbations in the expression of cytotoxic factors resulting in reductions in cytotoxic proteins required for effective lysis of viral and microbial particles. Importantly, NK cells have both activating and inhibitory receptors, successful killing of target cells occurs through integrated signalling by activating and inhibitory receptors, and co-receptors. Inhibitory receptors are activated through the recognition of MHC class I proteins and this inhibits NK cytoxic activity [35,36]. Activating receptors are important for eliminating tumours, and other microbes through intracellular signal transduction mechanisms that connect them to immunodominant tyrosine based activation motif (ITAMS) adaptor proteins [35-37]. Certain viruses can affect NK receptor signalling thus reducing cytoxic activity. For example, the cytomegalovirus viral genes can regulate NK inhibitory receptor expression preventing the induction of activating receptors [38]. An increase in viral load occuring during the course of CFS/ME, may trigger defective cytotoxic receptor activations hence resulting in compromises to NK cytotoxic activity. The significance of the overall decrease in cytotoxic activity at T3 is unclear.

The exact consequence of alterations in CD5^6bright^CD16^-^ NK cells in CFS/ME is not fully known, however, patterns of CD56^bright^CD16^-^ NK cells were affected by seasonal changes which may affect NK cytokine production. Incidentally gene expression of *IFNG* which is an important NK cytokine was significantly decreased in the NK cells of CFS/ME patients [25], which may be related to the decrease in CD56^bright^CD16^-^ NK cells. Decreases in CD56^bright^CD16^-^ NK cells have been observed in coronary heart disease, allergic rhinitis and juvenile rheumatoid arthritis while in diseases such as Chronic Obstructive Pulmonary Disease (COPD) CD56^bright^CD16^-^ NK cells have been reported to be increased [39,40].

IL-2, a pro-inflammatory cytokine produced by Th1 cells [41] is required for naïve CD4^+^T cell differentiation into Th2 and regulatory T cells (Tregs) in the presence of IL-4 and transforming growth factor beta (TGF-β) respectively [41]. Binding of IL-2 to its high affinity receptor IL-2R induces the proliferation of T cells and memory CD4^+^ and CD8^+^T cells [42-44]. It is also has important roles in generating effector functions for B cells, CD56^bright^NK and CD8^+^T cells [45]. IL-2 regulates Treg cells and interestingly, CD4^+^CD25^+^Foxp3^+^Treg cells, have been reported to be significantly increased in the CFS/ME patients in comparison to non-fatigued controls [25]. An increase in IL-2 may suggest a shift towards Th1/pro-inflammatory immune response in CFS/ME patients.

Anti-inflammatory IL-10 exerts inhibitory effects on cytokine secretion and impedes pro-inflammatory cytokine secretion by multiple cells including Th1 cells (IFN-γ), macrophages/monocytes (IL-1, IL-T2, IL-8, IL-12 and TNF-) and NK cells (IFN-γ and TNF-α) [46]. A decrease in IL-10 favours an increase in pro-inflammatory responses and this may increase the prevalence of Th1 like cytokines. IL-17A is expressed by Th17 cells, it recruits and activates neutrophils, stimulates the generation of pro-inflammatory cytokines, chemokines and increases antimicrobial gene expression [47-50]. IL-17A is therefore an important immunoregulator during microbial infections as it activates immune cells to secrete pro-inflammatory factors. A decrease in IL-17A may contribute to the prevalence of infections. A possible explanation for the observed changes in the secretion of this cytokine may be related to TGF-β which at optimal levels directly promotes IL-17A generation while reducing IL-2 [51]. Thus, in the CFS/ME patients, TGF-β may be decreased causing an increase in IL-2. Therefore, cytokine release in CFS/ME patients undergoes shifts during the course of the disease where patients may present with either an amplified or depressed anti-inflammatory or pro-inflammatory cytokine profile. These alterations in cytokine secretion may occur during the course of the disease and at different times causing either a shift towards a predominant Th1 or Th2 immune response in CFS/ME [25-27,52,53]. This makes it difficult to establish a unique CFS/ME-like inflammatory cytokine profile. The observed pattern of cytokine distribution among our CFS/ME patients is consistent with equivocal findings in the literature [54-58]. In adolescents with CFS/ME cytokine secretions have been observed to be correlated with seasonal variations [59]. Therefore, CFS/ME may be associated with oscillations in pro and anti-inflammatory cytokines, supporting the heterogeneity and multifactorial nature of the disease and the diversity in symptom presentations.

## Conclusion

In conclusion, altered regulation of immunological function, in particular reduced cytotoxic activity of innate immune cells, is a key component of CFS/ME. This longitudinal study has identified NK cytotoxic activity and possibly CD56^bright^CD16^-^ NK cells as potential biomarkers for diagnosing CFS/ME. The observation of immune dysregulation made in this study in relation to CFS/ME patients have been observed in various immunological diseases. This suggests the need for further investigations into the underlining disrupted mechanism of decreases in cytotoxic activity. It is important to note that these cytokine profiles were measured following mitogenic stimulation of PMBCs, serum measurements of cytokines may display different results. Further studies are therefore required to investigate whether changes in cytokine secretions from activated PMBCs and/or serum levels are associated with severity and progression of the complex clinical presentations in CFS/ME pathology.

## Competing interests

The authors have no competing interests.

## Authors’ contributions

EWB DRS KJA MVD SMG designed the experiments. EWB SBR JK SLH executed the experiments. EWB performed all data analysis. SMG KJA supplied the reagents. MVD collected and analysed the clinical data. EWB wrote the paper. MVD KJA SMG DRS DP LT critically reviewed the manuscript. All authors read and approved the final manuscript.
